# Supplementary material for: Comparisons of fatigue between dialysis modalities: A cross-sectional study
Source: PLoS One. 2021 Feb 10;16(2):e0246890. doi: 10.1371/journal.pone.0246890 (PMC7875388; doi:10.1371/journal.pone.0246890)
Supplement: Attachment — Submitted filename: Response to Reviewers PONE D 20 17573.docx [file pone.0246890.s002.docx]

Response to Reviewers

In particular, the aims of this study should be clarified (validation study versus assessment of fatigue across different dialysis modality), the use of POMS instead of SF-36 needs further justification and more emphasis should be given to the difficulties (and limitations) of interpreting results when using different measures of fatigue as presented in this study. The discussion should focus more on the outcome of fatigue then interventions to improve fatigue.

**RESPONSE: Thank you for raising these very important points. We have clarified the aims of this study in the Introduction as follows: “The aim of this cross-sectional study was to compare the prevalence or severity of fatigue among patients receiving different dialysis modalities, including HD, online HDF, PD, and combined therapy with PD and HD.” As we mentioned in the Limitation, we could not validate the effectiveness of our original scale of fatigue using this study design. Since the last sentence of the Introduction of "Additionally, the effectiveness of our original, very simple scale of fatigue was verified" is inadequate, we have deleted this sentence. We did not use SF-36, especially its the vitality scale, despite the frequently use in this area [Ref. 2, Ref. 3] and used POMS instead for two reasons. First, it includes the fatigue scale. Second, it is widely used in clinical practice and covered by the public medical insurance system in Japan. The associations between the scores of POMS, VAS and our original scale of fatigue were significant but weak. Additionally, the effects of several confounding factors, such as age, BMI, employment status and serum creatinine, on the presence or severity of fatigue differed depending on the scales. These discrepancies indicate the fact that different measures could yield different results. We have added this information to the Discussion. As suggested, we have deleted the discussion on the interventions for fatigue because this information is not necessary. We modified the discussion focused more on the outcome of fatigue.**

Please include your amended Funding Statement within your cover letter. We will change the online submission form on your behalf.

**RESPONSE: As suggested, we amended Funding Statement within our cover letter.**

Please include your updated Competing Interests statement in your cover letter; we will change the online submission form on your behalf.

**RESPONSE: As suggested, we updated Competing Interests statement in our cover letter.**

Reviewer #1: I read with interest the manuscript “Comparisons of fatigue between dialysis modalities: A cross-sectional study” by Maruyama and colleagues. This study provides interesting findings on fatigue in different dialysis modalities, as assessed through three different fatigue evaluation scales (POMS, VAS and an original scale).

Nevertheless, I have a few comments and questions to ask the authors. For simplicity, I will comment by sections.

METHODS

Subjects

1. Could the authors comment on how the patients were screened for inclusion in the study? Were all patients in the included centers screened for inclusion? Was there a minimum duration of dialysis for inclusion (eg. established on dialysis for at least 3 months)?

**RESPONSE: Thank you for raising this important point. As we described in the Materials and methods, we excluded patients under 18 years of age, in addition to patients with a mental disorder, such as schizophrenia, depression or dementia, and those with terminal cancer. We did not determine other inclusion or exclusion criteria and did not exclude patients with shorter duration of dialysis. Unfortunately, we are unaware of the detail of screening process at each center. We have added this information to the Limitation.**

2. The authors specify that “any patient that had a diagnosable mental health disorder or medical illness, including dementia and terminal cancer were excluded”. As patients with depression, CVD, diabetes, etc. are included in this study, it appears that the “diagnosable mental health disorder or medical illness” warranting exclusion from the study were more specific. The authors should further specify the exact exclusions and/or modify this statement in the manuscript.

**RESPONSE: Thank you for pointing out this error. As suggested, we did not exclude patients with history of CVD or diabetes. Therefore, the word of “medical illness” is inappropriate. We have corrected the description of exclusion criteria as follows: “Patients under 18 years of age, in addition to patients with a mental disorder, such as schizophrenia, depression or dementia, and those with terminal cancer were excluded.”**

Assessments of fatigue and depression

3. All used scales are very well described in the manuscript. However, no details are provided on how the responses to the self-reported questionnaires/scales were collected: were the patients completing the questionnaires in a written form by themselves or verbally answering to the questions to a member of staff? The authors should provide more details on the exact “self-reporting” procedure and if any exclusion from the study would have been related to these. For example, if the questionnaires were completed in a written format, were patients unable to write/read excluded or offered any help to complete the questionnaire?

**RESPONSE: Thank you for noticing this issue. We confirmed that all participants read and wrote questionnaires for themselves, although we did not excluded patients who need help to answer “written format” questionnaires. We noticed that there was a bias in recruiting participants for this study. We have added this information to the Materials and methods, the Results and the Limitation.**

Statistical analysis

4. Were the variables included in the multivariate analysis chosen on biological/clinical plausibility alone or was a univariate analysis done? As smoking was significantly different across groups, can the authors explain why it was not included in the multivariate analysis?

**RESPONSE: Thank you for raising this important point. On multivariate analysis, we selected several confounding factors reported to be associated with fatigue in dialyzed patients. However, there could be a possible confounding factor, including smoking status, as the reviewer suggested. Therefore, we changed the methods of variable selection. We also included confounding factor that had shown an appreciable association (P<0.1) with a measure of fatigue in multivariate analysis in order to avoid omissions error and created new Table 2 to 5. We have added this information to the Materials and methods and have modified the descriptions in the Results and Discussion, because the results of regression analysis were partially changed.**

RESULTS

5. Following on question #1, the authors should provide some information on the included cohort compared to the dialysis population in the participating centers. How many patients were screened for inclusion; how many were excluded and for which reason, etc? This might also need to be mentioned in the limitations of the study depending on the representativity of this cohort compared to the dialysis population screened.

**RESPONSE: Thank you for raising this important point. As described before, we are unaware of the detail of screening process at each center. Additionally, there could be a bias in recruiting participants for this study and representative sampling may not reflect the general dialysis population, because all participants read and wrote questionnaires for themselves although we did not excluded patients who need help to answer written format questionnaires. We have added this information to the Limitation.**

6. Can the authors comment on the predominantly male cohort: is this proportion representative of the dialysis population in Japan or is this related to higher consent to study by men or exclusion of more women due to exclusion criteria?

**RESPONSE: Thank you for this helpful comment. The percentage of male participants in this study was 69.1% and we think that it is similar to the general dialysis population in Japan. According to annual surveys of dialysis facilities throughout Japan in 2017, 65% of prevalent dialysis patients were male [Ref. 19].**

7. Could the authors provide a brief statement on modality choice in the participating centers (HD vs HDF vs PD vs combined HD/PD): patients’ choice, medical indication, ‘PD first’ policy, etc?

**RESPONSE: Thank you for this helpful comment. As we described in the Limitation, selection bias, especially physicians’ preference, for choice of dialysis modalities was considerable, because this study was not a randomized controlled study, but an observational study. PD First policy is recommended in order to maximize the advantages of PD therapy including excellent comparable survival, lower cost and improved quality of life also in Japan [Ref. 20, Ref. 21]. However, its long-term continuation is difficult because of a decline in residual renal function and deterioration of the peritoneal membrane. Patients who become unmanageable only by PD need to change the modality of RRT. Although they often directly transfer to HD, transfer to combined therapy with PD and HD, generally consists of 5-6 days of PD and commonly one HD session per week, was relatively common in Japan. PD therapy and combined therapy with PD and HD are thought to be performed especially among young and socially engaged patients. Indeed, the mean age of PD patients was 63.5 years, whereas the mean age of HD patients was 69.4 years [Ref. 19]. Additionally, the number of patients treated with online HDF have been increasing after the 2012 revision to the medical reimbursement system, and have reached 70000, approximately 30% of all dialysis patients at the end of 2017 in Japan. Since the mean age of HDF patients was 66.6 years and approximately 3 years younger than HD patients, this therapy is also thought to be performed especially among young and socially engaged patients [Ref. 19]. Therefore, we think that the prevalence or the severity of fatigue should be investigated not only among patients on PD and HD but those on online HDF and combined therapy with PD and HD. We have added this information to the Introduction and the Discussion.**

8. In the introduction, the authors mention that “It is unclear whether dialysis-related factors such as the type of dialysis modality and the adequacy or frequency of dialysis are associated with fatigue”. Were data on dialysis prescription (number of exchanges and volume of PD; treatment time and frequency on HD, etc) and on dialysis adequacy (Kt/v or URR) collected as part of this study? If so, these should be described and potentially included in the regression models. If these data were not collected, it should be mentioned in the limitation of the study as they could potentially be important confounders.

**RESPONSE: Thank you for noticing this issue. As the reviewer indicated, we did not have the data of neither adequacy nor frequency of dialysis. We have deleted these descriptions from the Introduction. Furthermore, we have added following sentences to the Limitations: “The effect of residual confounding factors including adequacy or frequency of dialysis and residual renal function, was unavoidable.”**

9. In the paragraph describing results included in Tables 2 and 3 (p.10-11), the same sentences appear twice. Table 2 describes the logistic regression model for highest score of fatigue (dichotomic outcome) and not the severity of fatigue. This should be corrected in the manuscript. In the second part of that paragraph (describing Table 3), the same sentence describing the association of employment status is used, mentioning the “lower risk of fatigue”. Here, it appears that this results should refer to a “lower severity/score of fatigue” (continuous outcome).

**RESPONSE: Thank you for this helpful comment. As suggested, we have modified the description of the Results. In the results of logistic regression analysis, we changed the description of outcome from the severity of fatigue to the presence of fatigue, because objective variables were not continuous but dichotomic variables. In addition, we changed the description of outcome from the risk of fatigue to the severity of fatigue in multiple regression analysis.**

DISCUSSION

10. Could the authors comment on the median VAS and POMS values obtained in their study compared to previous studies?

For example, in a Malaysian study comparing quality of life in HD vs CAPD, mean VAS scores were around 76-77 vs. 48 in the present study.

[Surendra NK, Abdul Manaf MR, Hooi LS, et al. Health related quality of life of dialysis patients in Malaysia: Haemodialysis versus continuous ambulatory peritoneal dialysis. BMC Nephrol. 2019;20(1):151. Published 2019 Apr 30. doi:10.1186/s12882-019-1326-x]

In contrast, a Polish study evaluating defense mechanism in dialysis patients found the fatigue subscale of the POMS around 5-11 vs. 11.3 in the present study.

[Nowak Z, Wańkowicz Z, Laudanski K. Denial Defense Mechanism in Dialyzed Patients. Med Sci Monit. 2015;21:1798-1805. Published 2015 Jun 22. doi:10.12659/MSM.893331]

The generalizability of the results from the present study should be mentioned in the discussion in light of such differences or similarities.

**RESPONSE: Thank you for this helpful comment. The scales of fatigue might differ from several sociodemographic factors such as age, gender and race. Unfortunately, we could not find the report comparing the scores of these scales between different populations. The VAS was graduated from 0 (worst imaginable health state) to 100 (best imaginable health state) in the Malaysian study the reviewer introduced, whereas it was graduated from 0 (no tiredness at all) to 100 (complete exhaustion) in our study. As indicated, the fatigue subscale of the POMS scores in the Polish study including 115 dialysis patients the reviewer introduced were lower than those of our study [Ref. 27]. On the contrary, these scores at baseline of randomized controlled trial including 15 HD patients in U.S. were approximately similar to the present study [Ref. 28]. We have added this information to the Discussion.**

FIGURE 1

11. In Figure 1, for the graph representing the results from the original scale, asterixis (*) are used but no legend for this is provided.

**RESPONSE: As suggested, we have added the explanation of asterixis to the Figure Legend.**

In conclusion, this manuscript presents very interesting findings on severity of fatigue in different dialysis modality, including the combined therapy of PD and HD, which is used much more frequently in Japan than elsewhere in the world. The cross-sectional, observational nature of the study, the potential for selection bias and residual confounding are limitations of this study, which have been clearly identified by the authors in the discussion.

**RESPONSE: Thank you for the favorable review you gave our manuscript.**

Please note that the data underlying the findings is described as fully available within the manuscript and its Supporting Information files on the submission but no supporting files were attached to the manuscript for review so I cannot comment on the “full availability” of the data.

**RESPONSE: As suggested, we attached data set we used to the revised manuscript.**

Reviewer #2: While fatigue is very important for patients on dialysis, I'm not sure that this study adds anything new to the existing body of literature on this topic. Furthermore, I have outlined some major methodological concerns below.

**RESPONSE: Thank you for the review you gave our manuscript. We wholly revised our manuscript according a number of helpful comments. As described before, the aim of this cross-sectional study was to compare the prevalence or severity of fatigue among patients receiving different dialysis modalities, including HD, online HDF, PD, and combined therapy with PD and HD. The main result was the prevalence and the severity of fatigue was not significantly different among patients receiving different dialysis modalities. The present study is the first study examined fatigue condition among patients receiving online HDF or combined therapy with PD and HD. We have added this information to the Introduction and Discussion.**

Aim - It is unclear whether the aim is to investigate the levels of fatigue across dialysis modalities or validate a new fatigue measure the authors have created

**RESPONSE: Thank you for raising this important point. We have clarified the aims of this study in the Introduction as follows: “The aim of this cross-sectional study was to compare the prevalence or severity of fatigue among patients receiving different dialysis modalities, including HD, online HDF, PD, and combined therapy with PD and HD.” As we mentioned in the Limitation, we could not validate the effectiveness of our original scale of fatigue using this study design. Since the description of "Additionally, the effectiveness of our original, very simple scale of fatigue was verified" is inadequate, we have deleted this sentence.**

Introduction:

- Second last paragraph: QOL and fatigue are not interchangeable so this paragraph seems a bit random – either explain how fatigue impacts QOL and thus is the research question for this study, or explore literature around fatigue instead

**RESPONSE: Thank you for raising this important point. We completely agree with the point that QOL and fatigue are not compatible. As suggested, we have deleted the description regarding the comparisons of QOL between different dialysis modalities. Instead, we focused on the clinical condition of fatigue.**

- Last paragraph: why would comparing modalities expected to help identify which subjects will benefit from interventions to reduce fatigue?

**RESPONSE: Thank you for this helpful comment. As suggested, we could not identify the subjects having benefit of interventions to reduce fatigue from this study design. We have deleted this sentence.**

Methods:

- I think the authors should justify why POMS was chosen for this study. If frequency of use was the basis, why wasn’t SF-36 used? POMS includes a subscale for fatigue but given that it is more of a symptom checklist-type measure with a focus on ‘moods’ than a symptom-specific questionnaire, it seems an odd choice of measure to which the authors’ measure is compared

**RESPONSE: Thank you for raising this important point. As mentioned before, we did not use SF-36, especially its the vitality scale, despite the frequently use in this area [Ref. 2, Ref. 3] and used POMS instead for two reasons. First, it includes the fatigue scale. Second, it is widely used in clinical practice and covered by the public medical insurance system in Japan. We have added this information to the Discussion.**

- What was the sampling methodology?

**RESPONSE: Thank you for this helpful comment. As mentioned before, we decided exclusion criteria, however we are unaware of the detail of screening process at each center. We have added following sentences to the Limitations: “We are unaware of the detail of screening process at each center. Additionally, there could be a bias in recruiting participants for this study and representative sampling may not reflect the general dialysis population, because all participants read and wrote questionnaires for themselves although we did not excluded patients who need help to answer written format questionnaires.”**

- The readers would benefit from greater details about the ‘original measure’. How was it developed/through what process? Has the content validity been established? Is this the first time that it has been used in a study? Why/how were grades 3, 4 and 5 chosen as ‘fatigued states’? Is this measure described else where in the literature? If so, please add in the relevant citation

**RESPONSE: Thank you for raising this important point. As we mentioned in the Materials and methods, we developed our original scale of fatigue and have already used in the previous two studies (Ref.14 and Ref. 15). However, only this scale was used for the assessment of fatigue in these two studies. It is first time to compare the findings of this scale with those of well-established scales and we found that the scores of the POMS, VAS, and our original scale of fatigue were significantly inter-related. However, convergent and discriminant validity, test-retest reliability, or social desirability could not be clarified using this study design as we mentioned in the Limitation. As described in the Materials and methods, our original scale of fatigue is a five-grade evaluation including Grade 1 (inexhaustible, feels well, and extremely active), Grade 2 (tireless, acts in the ordinary way without a sense of fatigue), Grade 3 (mild fatigue, acts in the ordinary way but feels tired), Grade 4 (moderate fatigue, feels tired with light work), and Grade 5 (intense fatigue, very tired, and falls asleep). Therefore, we defined Grades 3 to 5 as fatigued state because the examinee realizes the presence of mild, moderate or intense fatigue.**

- Depression needs to be better integrated into the rationale of this study

**RESPONSE: Thank you for this helpful comment. As we mentioned in the manuscript, it is well-known that depression, the most important psychological factor, is strongly associated with fatigue and they are closely interrelated. Although exclusion criteria included the patients with depression, we could not exclude the effects of depressive status on the prevalence or severity of fatigue. Therefore, we assessed the presence and severity of depressive symptoms using the BDI–II and conducted sensitivity analyses including only patients with lower score of BDI-II. Since the results of these sensitivity analysis were similar to the results including all subjects, depressive state was thought to have a low impact on the prevalence or severity of fatigue in our study. We have added this information to the Results and Discussion.**

Results:

- Given that POMS, VAS and the original scale were all used to explore fatigue, the rho seems quite low. explore this in the discussion

**RESPONSE: Thank you for this helpful comment. As suggested, the associations between the scores of POMS, VAS and our original scale of fatigue were significant but weak. Additionally, the effects of several confounding factors, such as age, BMI, employment status and serum creatinine, on the presence or severity of fatigue differed depending on the scales. These discrepancies indicate the fact that different measures could yield different results. We have added this information to the Discussion.**

- POMS and VAS did not differ across four groups, but the original scale did – what does this tell us about the fatigue levels across these groups? Or what does this say about the validity of the original scale?

**RESPONSE: Thank you for this helpful comment. Although only the score of our original scale of fatigue was significantly different among patients on different dialysis modalities in univariate analysis, this finding disappeared in multivariate analysis. Therefore, we considered that the severity of fatigue assessed by our original scale of fatigue were not different. As mentioned before, we could not validate the effectiveness of our original scale of fatigue using this study design.**

Discussion

- Discrepancy in results in existing literature regarding fatigue may also be due to the fact that measures of fatigue vary across these studies (as evident in this study where different measures yield different results)

**RESPONSE: Thank you for this helpful comment. As suggested, the effects of several confounding factors, such as age, BMI, employment status and serum creatinine, on the presence or severity of fatigue differed depending on the scales. Additionally, the associations between the scores of POMS, VAS and our original scale of fatigue were significant but weak. These discrepancies indicate the fact that different measures could yield different results. We have added this information to the Discussion.**

- Discussion on the interventions for fatigue seems out of place – link the results of this study back to what the implications are for future research aiming to look at interventions for fatigue across different modalities

**RESPONSE: Thank you for this helpful comment. We completely agree with the opinion that discussion on the interventions for fatigue was not necessary. As the Editor also pointed, we deleted the description of this discussion and focused more on the outcome of fatigue.**

- 4th limitation is noted as the fact that ‘convergent and discriminant validity, test-retest reliability or social desirability could not be clarified using this study design’. This is a major flaw of this study, as using a measure that has not been adequately validated runs the risk of yielding not only inconclusive but also misleading results. I would advise that the authors rethink the true aim of this study

**RESPONSE: Thank you for raising this important point. As mentioned before, the aim of our study was to compare the prevalence or severity of fatigue among patients receiving different dialysis modalities, including HD, online HDF, PD, and combined therapy with PD and HD. We could not validate the effectiveness of our original scale of fatigue using this study design. We have clarified the aims of this study in the Introduction. In this study, we employed our original scale of fatigue as a complementary test for direct patient-reported outcome, because it is easy to use, and its simplicity free usage. We used this test both dialysis and dialysis-free day and found that there were significant differences. The result may indicate the different diurnal and weekly profiles of fatigue between HD and PD, despite the equivalent levels of fatigue on dialysis day in PD and HD. We added this point in the revised manuscript, and we think this needs to be clarified in future study by using POMS, VAS and SF-36.**
