# Supplementary material for: Comparisons of fatigue between dialysis modalities: A cross-sectional study
Source: PLoS One. 2021 Feb 10;16(2):e0246890. doi: 10.1371/journal.pone.0246890 (PMC7875388; doi:10.1371/journal.pone.0246890)
Supplement: Attachment — Submitted filename: Response to Reviewers PONE D 20 17573 20210114.docx [file pone.0246890.s004.docx]

Response to Reviewers

The suggested minor revisions are included in the edited tracked version attached and relate predominantly to the conclusion of the abstract and the manuscript. Further minor edits and grammatical corrections have been made throughout the manuscripts.

RESPONSE: Thank you for revising our manuscript. As suggested, we demonstrated data on age, gender and the proportion of diabetes only in the Result section. We revised the word from “native dialysis modality” to “preferred dialysis modality” in the third paragraph of the Discussion section. Additionally, we agree with the rephrased sentence in the last of this paragraph. We reformatted the list of references.
