# Supplementary material for: Comparisons of fatigue between dialysis modalities: A cross-sectional study
Source: PLoS One. 2021 Feb 10;16(2):e0246890. doi: 10.1371/journal.pone.0246890 (PMC7875388; doi:10.1371/journal.pone.0246890)
Supplement: Attachment — Submitted filename: Response to Reviewers PONE D 20 17573 20210126.docx [file pone.0246890.s006.docx]

Response to Reviewers

Suggested revisions have been attached (Edited revision 3) to ensure the conclusions are supported by the data (please note the current version implies a causal relationship rather than an association). Comments have been added in the manuscript version attached to outline further minor revisions including the limitation of the small sample size and improvement of the first paragraph in the discussion to reflect a concise summary of the findings. Without these amendments, the manuscript does not fulfil the publication standards. The editor encourages the authors to address the suggested edits and revisions.

RESPONSE: Thank you for revising our manuscript. As suggested, we amended our manuscript. First, we modified conclusion both of abstract and text to avoid any mention of causal relationship. Second, we modified the first paragraph of the Discussion. Third, we added limitation regarding the small sample size. Additionally, we approved all modifications.
